# Steroid-Induced Hiccups in a Patient Managed for Pseudo Foster-Kennedy Syndrome: A Case Report of Good Outcome With the use of Gabapentin

**DOI:** 10.7759/cureus.12893

**Published:** 2021-01-25

**Authors:** Ejike Egbu, Chidi Ihemedu, Ugochukwu A Eze, Chukwuemeka Nwajei, Morgan Ikponmwosa

**Affiliations:** 1 Ophthalmology, Lily Hospitals Limited, Warri, NGA; 2 Ophthalmology, Katsina Eye Center, Katsina, NGA; 3 Ophthalmology, Central Hospital, Sapele, NGA; 4 Radiology, Lily Hospitals Limited, Warri, NGA

**Keywords:** steroids induced hiccups, pseudo foster-kennedy syndrome, methylprednisolone, chlorpromazine, a case of pseudo foster kennedy syndrome, management of optic neuritis in a patient with diabetes, mechanism and pathway for hiccups, gabapentin as treatment for hiccups

## Abstract

The use of IV methylprednisolone has been shown to be associated with some adverse effects. The most feared side effect is acute gastrointestinal perforation and accelerated hypertension particularly during pulse therapy. Hiccups occur less frequently but can cause high levels of discomfort to the patient. In intractable cases, respiratory arrest and death can occur. This article reports the occurrence of hiccups in a patient managed for pseudo Foster-Kennedy syndrome. The hiccups were observed shortly after IV methylprednisolone was administered to the patient and abetted over a period of one week after it was discontinued. Hiccups occur through the neuronal pathway of the hiccup reflex arc, comprising the vagus nerve, phrenic nerve, parts of the sympathetic nervous system (T6-T12), and efferent fibers from the phrenic nerve that supply the glottis and the accessory muscles of respiration. The hiccups resolved with the use of gabapentin. This case report aims to add to the existing body of knowledge of the efficacy of gabapentin in the management of hiccups.

## Introduction

Hiccups also known as singlitus occurs due to an involuntary contraction of the diaphragm and the intercostal muscles leading to the inspiration of air, followed by a sudden closure of the vocal cords, producing the “hiccup” sound [1]. They can be seen as an unusual physiological response which is triggered by air movement [2]. Hiccups can occur due to disorders that affect the central nervous system, irritation of the vagus nerve, irritation of the diaphragm, metabolic disorders, surgery, infections, drugs, stress and anxiety, and idiopathic factors [3]. The presence of a space occupying lesions in the brain alongside with stroke can serve as a central cause of hiccups while peripheral causes can include: myocardial ischemia, gastroesophageal reflux disease, herpes infection, etc.

Hiccups are not predominantly found in adults; they can also occur in infants and children. In these cases, hiccups occurrence is usually referred to as self-limited episodes which are due to irritations caused by overeating, eating too fast, immediate change in ingested food temperature, drinking carbonated drinks, etc. These cases abate easily without any clinical significance [2]. However, hiccups can be triggered by changes in metabolic and endocrine conditions of patients [4].

The neuronal mechanism of hiccups is made up of afferent fibers of the vagus nerve, phrenic nerve, parts of the sympathetic nervous system (T6-T12), and efferent fibers from the phrenic nerve that supplies the glottis and the accessory muscles of respiration. The hiccup reflex center is thought to be a spinal reflex originating from C3-C5 segments of the spinal cord or a brain stem reflex originating from an area near the respiratory inhibitory centers in the midbrain [5-6]. Hiccups can be classified based on duration into acute or transient (less than 48 hours), persistent (longer than 48 hours), or intractable (longer than one month) [7]. Structural or functional irritation that involves the reflex arc can be seen in patients with intractable hiccups and these prolonged hiccups could cause weight loss, dehydration, fatigue, and even death in severe cases [2]. Although the prevalence of hiccups is equal in males and females, intractable hiccups are mostly seen in males [7].

The drugs that are known to cause hiccups in patients include steroids, benzodiazepines, barbiturates, antibiotics, phenothiazines, opioids, and alcohol. Steroids such as prednisolone, methylprednisolone, dexamethasone, oxanodrolone, and progesterone have also been implicated [7-8].

Treatment options for hiccups include the use of pharmacologic and nonpharmacologic agents if its cause can be established. Presently, chlorpromazine is the only drug approved by the Food and Drug Administration (FDA) for the treatment of hiccups because there are no large-scale studies on the efficacy of other regimens for this condition. However, undesirable results have been reported. Other medications such as gabapentin alone (which was used in this report) and baclofen have yielded positive results in the treatment of chronic hiccups [7, 9].

## Case presentation

**Figure 1:**
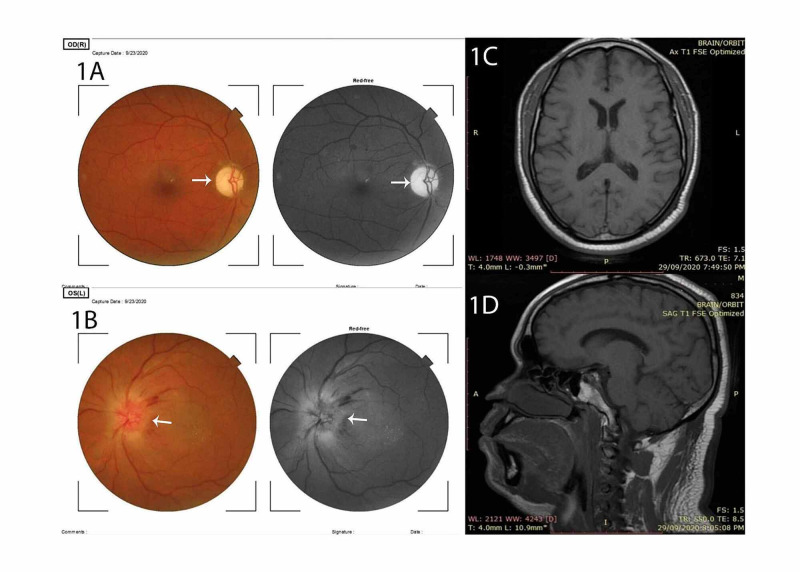
Pane 1A shows the fundus photographs of the right eye while 1B represents the left eye. Panes 1C and 1D show the MRI of the brain. The optic disc of the right eye is markedly pale while that of the left is markedly edematous and elevated, indicated by the white arrows. There is no intracranial mass in the MRI of the brain and orbit.

We present a 44-year-old insulin-dependent diabetes mellitus patient who presented with complaints of painless marked deterioration of vision in the right eye of one week prior to presentation. Ocular examination revealed visual acuity of 6/9 in the right eye and hand movement in the left eye. Marked optic disc palor in the right eye and disc edema in the left eye were shown in the optical coherence tomography (OCT). There were also macular exudates, and retinal hemorrhages, see Figure 1A,B. These findings were in keeping with diabetic papillopathy, a known cause of pseudo Foster-Kennedy syndrome. MRI of the brain and orbits did not show the presence of an intracranial tumor, see Figure 1C,D. He was scheduled for steroid management in line with the optic neuritis treatment trial protocol. On administering the first dose of IV methylprednisolone, he developed hiccups after about two hours. It worsened in frequency up to 20 hiccups per minute. The hiccups were severe and caused him chest pain and difficulty while speaking. It was abetted with the administration of gabapentin 300 mg tablet daily over a period of one week. During this period, the drug was well tolerated as no adverse effects were noted. He was further managed with oral prednisolone for the diabetic papillopathy which resolved after six weeks.

## Discussion

The management of hiccups is challenging due to the lack of sufficient evidence in the use of pharmacologic and nonpharmacologic agents [3]. Chlorpromazine is an FDA drug for persistent hiccups; however, Eze et al. reported that the use of metoclopramide and chlorpromazine was ineffective in controlling hiccups resulting from the use of dexamethasone in two patients [10-11]. Drugs such as gabapentin, baclofen, and metoclopramide have also shown promising results in managing persistent hiccups. Acupuncture has also been put forward as a nonpharmacologic intervention [1, 11]. In a study of antiemetic corticosteroid rotation from dexamethasone to methylprednisolone to prevent dexamethasone‐induced hiccup in cancer patients treated with chemotherapy, it was found that initial administration of dexamethasone was associated with a higher severity of hiccups compared to a re-administration, thus suggesting a possible increased tolerance to dexamethasone after initial dose [11]. In our patient, it was also observed that after the initial administration of IV methylprednisolone, he did not experience hiccups with the use of prednisolone tablets over the treatment period. This observation could be because our patient developed increased levels of tolerance to steroids or due to the effects of gabapentin. A study among Koreans aimed at preventing vomiting and nausea in individuals undergoing chemotherapy found that rotating dexamethasone to methylprednisolone was a viable method of preventing hiccups [8].

Gabapentin has been shown to be effective in managing intractable hiccups [12]. The mechanism by which gabapentin decreases hiccups is thought to involve the inhibition of the voltage-gated calcium channels in a subset of excitatory and inhibitory presynaptic terminals. The exact mechanism of binding is however unclear. It is also postulated that gabapentin has inhibitory effects on the diaphragm and other muscles of inspiration by modulating the endogenous release of gamma butyric acid (GABA) and glutamate [12-13].

A study by Porzio et al. [14] reports a five year experience with the use of gabapentin in the treatment of hiccups in patients with advanced cancer. It was found that 31 out of 37 in-hospital patients had a complete resolution of hiccups while four out of six experienced a reduction after being observed at home. However, the intensity of the hiccups increased in two patients.

Also, Campbell et al. [15] reports a case of an 86-year-old man who developed hiccups prior to admission to the hospital for the treatment of urinary tract infection. Metoclopramide was administered to the patient but there was no significant improvement after two days of usage. However, gabapentin was recommended based on his probable neurogenic etiology and there was a significant improvement in his hiccups within some hours, followed by a complete resolution on the second day of gabapentin treatment. Three days later, the hiccups reoccurred after he was readmitted for the treatment of pneumonia. The usage of metoclopramide also seemed abortive and gabapentin was reinitiated for the treatment of the hiccups which yielded success.

Li et al. [4] reported a case of intractable hiccups in a 55-year-old woman who developed diabetic ketoacidosis and thyroid storm. In their finding, the use of metoclopramide, omeprazole, hydrotalcite, domperidone, promethazine, chlorpromazine, acupuncture, continuous infusion of traditional herbal drugs, esmolol, and rhubarb enema was no effective in controlling the condition. The hiccups worsened and the patient developed respiratory failure and artificial ventilation was initiated which brought the hiccups under control.

Although other agents can be used in the treatment of steroid-induced hiccups gabapentin has been proven to yield remarkable results and more reports are needed to support this finding.

## Conclusions

Steroids are used in the management of several medical conditions and predispose patient to side effects such as acute gastrointestinal perforation and accelerated hypertension, particularly during pulse therapy using high-dose IV methyprednisolone, so clinicians should pay attention to these adverse side effects while treating patients with steroids. In this case report, the adverse effect of IV methyprednisolone administration was hiccups which was abetted with the use of gabapentin; we therefore propose the use of this drug for the control of hiccups.
